# Estimation of cumulative amplitude distributions of miniature postsynaptic currents allows characterising their multimodality, quantal size and variability

**DOI:** 10.1038/s41598-023-42882-9

**Published:** 2023-09-20

**Authors:** Susanna Gordleeva, Yulia Dembitskaya, Victor Kazantsev, Eugene B. Postnikov

**Affiliations:** 1 Scientific-Educational Mathematical Center “Mathematics of Future Technologies”, Lobachevsky State University of Nizhny Novgorod, Nizhny Novgorod, Russia; 2 Shemyakin-Ovchinnikov Institute of Bioorganic Chemistry, Russian Academy of Sciences, Moscow, Russia 117997; 3 Kursk State University, Kursk, Russia; 4 Neuroscience Research Institute, Samara State Medical University, Samara, Russia 443079

**Keywords:** Synaptic transmission, Data processing, Biophysical models

## Abstract

A miniature postsynaptic current (mPSC) is a small, rare, and highly variable spontaneous synaptic event that is generally caused by the spontaneous release of single vesicles. The amplitude and variability of mPSCs are key measures of the postsynaptic processes and are taken as the main characteristics of an elementary unit (quantal size) in traditional quantal analysis of synaptic transmission. Due to different sources of biological and measurement noise, recordings of mPSCs exhibit high trial-to-trial heterogeneity, and experimental measurements of mPSCs are usually noisy and scarce, making their analysis demanding. Here, we present a sequential procedure for precise analysis of mPSC amplitude distributions for the range of small currents. To illustrate the developed approach, we chose previously obtained experimental data on the effect of the extracellular matrix on synaptic plasticity. The proposed statistical technique allowed us to identify previously unnoticed additional modality in the mPSC amplitude distributions, indicating the formation of new immature synapses upon ECM attenuation. We show that our approach can reliably detect multimodality in the distributions of mPSC amplitude, allowing for accurate determination of the size and variability of the quantal synaptic response. Thus, the proposed method can significantly expand the informativeness of both existing and newly obtained experimental data. We also demonstrated that mPSC amplitudes around the threshold of microcurrent excitation follow the Gumbel distribution rather than the binomial statistics traditionally used for a wide range of currents, either for a single synapse or when taking into consideration small influences of the adjacent synapses. Such behaviour is argued to originate from the theory of extreme processes. Specifically, recorded mPSCs represent instant random current fluctuations, among which there are relatively larger spikes (extreme events). They required more level of coherence that can be provided by different mechanisms of network or system level activation including neuron circuit signalling and extrasynaptic processes.

## Introduction

Short-term synaptic plasticity is a key mechanism for information processing in the CNS, while learning and memory can be formed through long-term synaptic modifications. One of the basic neurophysiological methods used to investigate the alterations in synaptic transmission is analyzing the dynamics of inward postsynaptic membrane currents (PSCs). Proper analysis of the postsynaptic response amplitude fluctuations can quantify functional synaptic characteristics, reveal the locus of expression of synaptic modulation (pre- or postsynaptic site) and identify the mechanism that produces that modulation. However, this has proven difficult in practice due to the high trial-to-trial variability of experimental PSC recordings, which arise not only from instrumental noise but also from specific statistical features characterising underlying microscopic biophysical processes and details of their experimental registration.

Quantal theory of transmitter release was developed from the observation of compliance between the incremental amplitude of synaptic response fluctuations and the amplitude of spontaneous miniature synaptic events. Quantal synaptic theory has been widely used to study the functions of synaptic transmission and plasticity. A conventional model describing stochastic quantal transmission in synapses is called the binomial model^1^. This model assumes that the presynapse contains *N* potential sites for vesicle release with identical probability of release *p*. After presynaptic spike, the postsynapse receives neurotransmitter from number of vesicles *k* discharging following a binomial distribution:

1 $$\begin{aligned} P_{Bin}(k,N,p)=\left( {\begin{array}{c}N\\ k\end{array}}\right) p^{k}(1-p)^{N-k}, \end{aligned}$$

where $$\left( {\begin{array}{c}N\\ k\end{array}}\right)$$ is the binomial coefficient representing the number of combinations in which *N* sites can emit *k* quanta. Each vesicle release induces generation of a quantal current *q*. A general observed PSC is equal to $c=qk+\varepsilon$, where the transmission is considered as affected by the noise $\varepsilon$ normally distributed with the variance $\sigma ^2$. Thus, the distribution of PSCs is given by:

2 $$\begin{aligned} P(c)=\sum \limits _{k=0}^N P(c|k)P(k). \end{aligned}$$

Therefore, in theory, the amplitude of the PSCs varies by an integer multiple of an elementary quantal unit. Quantal size *q* corresponds to the distance between the narrow peaks in the amplitude histogram of the PSC and can be measured directly by the average amplitude of the miniature PSC (mPSC)—a uniquantal synaptic event caused by spontaneous vesicle release^2,3^. Respectively, the mPSC represents a key measure of postsynaptic processes in convectional quantal analysis, while noise simply plays the role of disturbances “blurring” the peaks registered in practical measurements.

Thus, three functional parameters *N*, *p* and *q* describe the synaptic transmission and define the synapse strength. Wherein, *p* and *q* represent presynaptic and postsynaptic efficacy, respectively. Synaptic plasticity will modulate these parameters. And the mechanism underlying an alteration of synaptic transmission can therefore be studied by monitoring changes in these parameters. These three synaptic parameters can be extracted from the distribution of PSC amplitudes. Many techniques have been developed for an analysis of the fluctuations in the evoked synaptic response. Among the most widely used methods are those based on deconvolution^4,5^ or convolution^6^ approaches, fluctuation analysis^4^, and variance-mean analysis^7^ (for review, see^8^). But they all require accurate estimation of quantal size and its variability from a histogram of mPSC amplitude data and fail when quantal response is small relative to the recording noise. In practice, it has been challenging to extract the size of a quantal response from mEPSC amplitude distributions due to strong quantal-size variability and the measurement noise.

Consideration based on the traditional quantal theory, as described above, is adequate for the evoked PSCs that cover a significantly wide range of current amplitudes, up to hundreds of pA when multiple quantal peaks are clearly exhibited. On the contrary, the consideration of spontaneous microcurrents of a few dozen of pA, when one operates in the vicinity of one quantum range reveals a drastically different picture: the respective distribution is practically reduced to a single skewed bell-shaped curve that contradicts the conventional approach to the representation of the p.d.f. as a combination of a point-wise supported single bar-like discrete components dispersed by the convolution with the Gaussian function^3,9–14^.

An experimentally realistic picture of mPSCs recordings resulting in a practically continuous asymmetric distribution due to a combination of a high variability of the quantal response (biological noise) and the measurement noise^15^. Such a distribution can be formed by a single quantum released by one studied synapse with the size about 17.4 pA and from neighbouring synapses with quantal sizes of 7–12 pA^16^. The main origins of mPSC amplitude variability are vesicle size, neurotransmitter content, multivesicular release, neurotransmitter release mode, electronic distance from recording site, saturation of postsynaptic receptors and intrasynaptic variation in receptor density^3,17^.

As mEPSCs for an ensemble of single events are quantal responses, their distributions should be unimodal with the mode corresponding to the typical quantum. However in some cases, high mEPSC variability arises due to the presence of obscure multimodality in mEPSC amplitude distributions. Multimodal mEPSC amplitude distributions result from the near-synchronous release of variable number of vesicles and have been demonstrated at central excitatory and inhibitory synapses^10,16,18–20^. Multimodal mEPSC amplitude distributions may occasionally arise from cells at some stages during regeneration or development. In such cases, small subminiature potentials may correspond to the developing junctions. The subminiature PSCs do not seem to be related to the unit of evoked release (this cannot be used in the determination of quantal response); which can be described by the major mode of larger spontaneous mEPSCs. However, clear observing two or more peaks on the mEPSC amplitude histograms is quite rare due to the strict requirements for accurate measurements. More often, the amplitude distributions of miniature events become smeared by cable effects. The facts described above motivate the development of statistical techniques for robust rigorous determination of quantal response through precise analysis of mPSCs data and adequate interpretation of the obtained results^21^.

Despite several, rather unordered trials to use different skewed unimodal functions for approximation of the mPSCs amplitude distributions, such as Gamma distribution^11,22–24^, the Weibull distribution^11,25,26^, the Gumbel function^27^, there has been no sequential, convenient, and robust statistical model for precise description of the shape of mPSC distributions based on the specificity of processes occurring around the threshold of microcurrent excitation amidst the fluctuating environment of the comparable noise range^3,14,21,27^.

Thus, we can stress the need for more precise statistical analysis of mPSCs distributions when microcurrents’ magnitudes are comparable with the typical range of magnitudes of spontaneous noisy contributions, which prevents a deconvolution of both processes and requires building a model for the fluctuating process considered as a whole.

Here, we present a sequential procedure for precise analysis of mEPSC amplitude distributions. To illustrate the developed approach, we chose previously obtained experimental data of mEPSCs amplitudes on the effect of the extracellular matrix on synaptic plasticity^28^. Proposed statistical treatment allowed us to identify previously unnoticed additional modality in the mEPSC amplitude distribution, indicating the formation of new immature synapses upon ECM attenuation. We show that our technique can reliably detect multimodality in the distribution of mEPSC amplitude, allowing for accurate determination of the size and variability of the quantal synaptic response. Thus, the proposed approach can significantly expand the informativeness of both existing and newly obtained experimental data.

## Theory and results

To illustrate the proposed approach, we use whole-cell patch-clamp recordings of mEPSCs which were obtained from CA1 pyramidal neurons of young mice (4–6 weeks old)^28^. This study^28^ examined the role of brain extracellular matrix in synaptic plasticity. Hippocampal slices were treated with either chondroitinase ABC (ChABC), which induces enzymatic attenuation of ECM, or a sham solution and were used for mEPSCs recordings. To reveal NMDA receptor mediated current component mEPSCs were recorded in Mg$^{2+}$ free extracellular solution in the presents of AMPA receptor blocker NBQX. NMDA receptor-mediated mEPSCs were measured for both cases: sham solution and ChABC-containing solution. mEPSCs were measured after blocking action potentials with tetrodotoxin to insure that there is no release due to spontaneous action potentials.

Accordingly, for analysis we use four mEPSCs datasets recorded under different conditions: (1) sham extracellular solution (SHAM), (2) Mg$^{2+}$ free sham solution (SHAM 0 Mg), (3) solution containing ChABC (ChABC), (4) ChABC-containing Mg$^{2+}$ free solution (ChABC 0 Mg). The recording data represent average amplitudes within 30 s equal subsequent time intervals. Each combined dataset includes data recorded from 6 individual cells, and their resulting lengths are 120, 120, 245, and 254 data points respectively.

**Figure 1:**
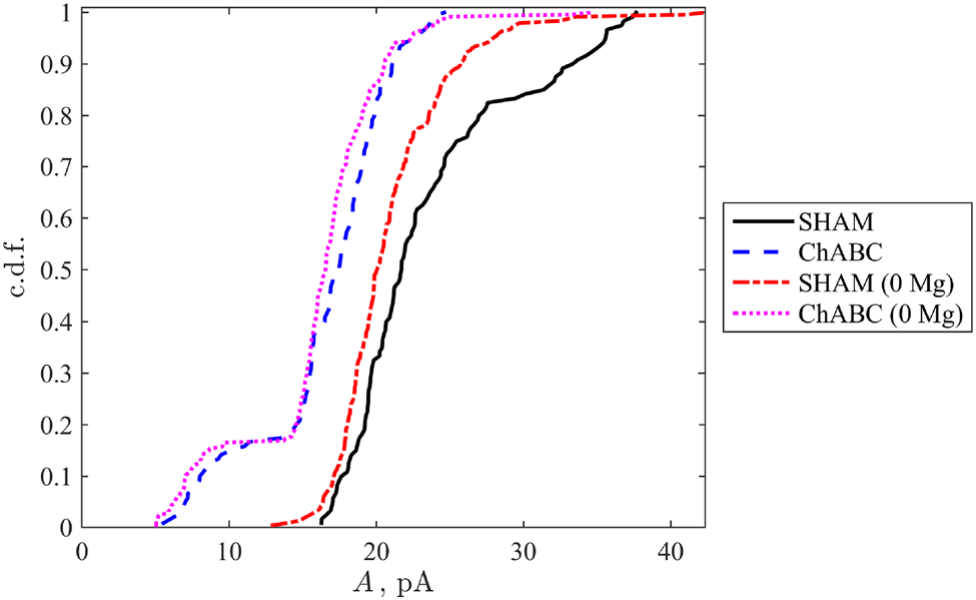
Empiric cumulative probability functions (c.d.f.) for the studied mEPSCs datasets.

To reveal the type of a probability distributions corresponding to all data samples, the standard robust statistical approach^29^, operating with the empiric cumulative distribution function, was applied. The empirical cumulative distribution functions were built as follows: the amplitudes $A_n$ (*N* in total)were sorted in ascending order and the discrete values of the cumulative distribution function equispaced varying from 1/*N* to 1 were assigned as ordinates corresponding to $A_n$’s, see Fig. 1.

Evidently, three out of four cases (i.e. except for SHAM (0 Mg)) represent two-stage probabilistic processes characterised by a plateau at intermediate values of mEPSC amplitudes followed by the final achievement of a plateau at large amplitudes. This implies that there exists a type of bimodal probability distribution density $P(A)=d(c.d.f)/dA$.

Focusing on a general procedure rather than testing a set of arbitrary chosen distributions, we consider the following representation

3 $$\begin{aligned} P(A)=r\cdot c.d.f.(A)\left( 1-\frac{c.d.f.(A)}{K}\right) ^n, \end{aligned}$$

which resembles the approach known in the populations dynamics as the Richards equation^30^—a very flexible tool for the approximation of sigmoidal curves; it also takes into account that $P(A)=d(c.d.f(A))/dA$. Equation (3) also satisfies two exact statistical conditions: when $c.d.f.(A)=0$, the probability density $P(a)=0$, and when the cumulative distribution function tends to the saturation (we left $K=1$ in Eq. (3) for the sake of convenience of the experimental data processing. Here $c.d.f.=1$ asymptotically taking into account weak second component localised at large amplitudes, i.e. this $K\ne 1$ corresponds to the asymptotics of one principal studied component). The power-law index *n* allows reproducing significant variability in asymmetry of the *c*.*d*.*f*. and, respectively, skewness of the *P*(*A*).

Note also the similarity of Eq. (3) and the probability density function of the particular case of the binomial distribution Eq. (1) for $K=1$ (but it can be easily rescaled for $p\rightarrow p/K$). Although its clear that the binomial distribution is not applicable to mPSC in contrast to PSC, the formal form representing a product of the current value (i.e. the discharge process) and its complementary (i.e. an absence of the discharge process) is quite general for characterising random processes with alternative with alternative outcomes. As noted above, operations with mPSCs involve one quantal size, i.e. $k=1$; $n=N-1$. Therefore, by using Eq. (3) we consider not a fixed *p* (that is the case of conventional approach to studying large quantized PSC), but a fluctuating ones reflected in the registered mPSCs.

This approach does not require hypothetical judgement and does not depend on whether a squared deviation from a set of trial curves or level of some probability-comparing statistical criterion is enough. To test this approach according to the illustrative criterion, we use the method based on the generalised Fisher–Pry transformation, which provides linearization of solutions to Eq. (3) via the following non-linear variable alteration

4 $$\begin{aligned} FP(A)\equiv -\ln \left[ \frac{1}{n}\left( \frac{K}{A}\right) ^{n-1}\right] =rA+{A_{m}} \end{aligned}$$

for a sequential set of the values of the parameter *n*. Note that we consider the general Richard’s case when *n* can be non-integer too. From the statistical point of view, the binomial distribution goes to the Beta-distribution and, in the limiting case of $n\rightarrow 0$, to the Gumbel distribution.

**Figure 2:**
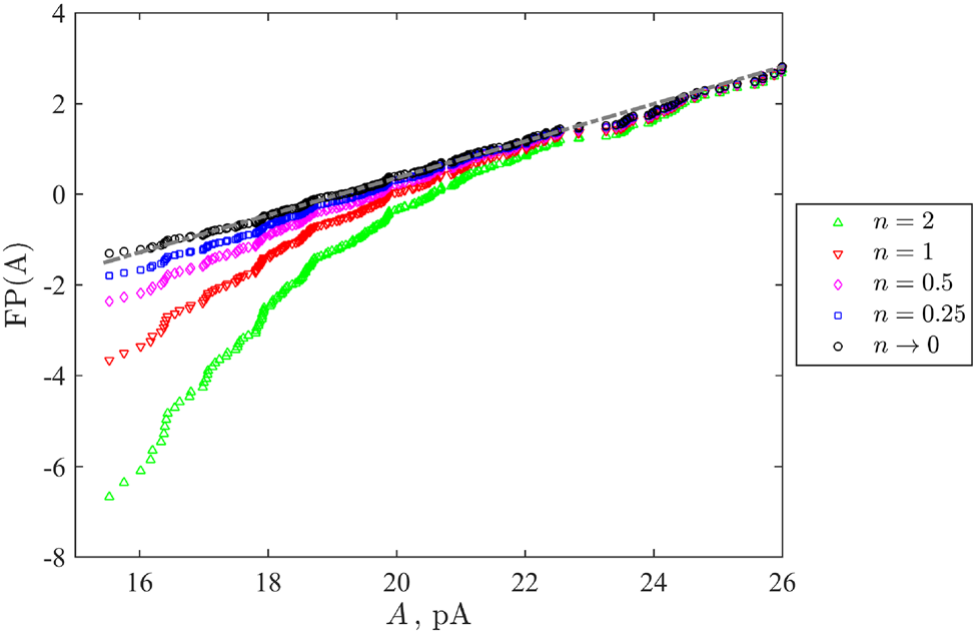
Recorded in Mg$^{2+}$ free sham solution (SHAM 0 Mg) experimental data points of mEPSCs sorted in the ascending order and plotted in the generalised Fisher–Pry form (4) with different values of the parameter *n* (same as in Fig. 3D–F). The grey dash-dotted straight line highlights the fitted linearization as in Fig. 3F.

Figure 2 illustrates our approach by the case study of the (SHAM 0 Mg) data, which does not show a clearly exhibited bi-component structure, i.e. we can operate with the whole data sequence. Integer values of *n* do not lead to linearized plots completely. This means that we can not consider the analysed process as a successful release of a quantum among several total trials occurring with different probability. Diminishing *n* (as fractional indices) leads to more and more straightened point distributions, which achieve the acceptable linearization in the asymptotic case $n\rightarrow 0$, i.e. the Gumbel distribution.

Next, we reveal two components in p.d.f. for SHAM, ChABC 0 Mg, ChABC experimental conditions and illustrate applicability of the Gumbel statistics to considered mEPSC datasets. For this, we use an approach called Loglet decomposition, which was developed^31^ for modelling growth processes comprised of two or more subprocesses of the same nature. This online tool is available at !https://logletlab.com/! and as of recently allows operating with main principal sigmoidal growth models including general Richard’s and Gompertz’s models. The key idea behind this approach is attempting to fit such a growth curve by a successive sequence of components and to achieve the best approximation by minimising some objective functions supplied with a set of representations of the components as linear functions. These functions can be obtained by an appropriate co-ordinate transformations.

Although the Loglet approach was initially developed for the data-based parameter estimation of dynamical systems describing growth with saturation, it can be naturally transferred to the problem of the identification and decomposition of the statistical properties of a given distribution of mEPSC amplitudes. This transferability is based on the following similarity: (1) the cumulative distribution function represents the monotonously growing saturation value equal to unity, which makes it a mathematical equivalent to the growth functions used within the frames of Loglet approach; (2) the mEPSC amplitudes *A* are strictly positive quantities, and the exact $c.d.f.\rightarrow 1$ when $A\rightarrow \infty$ that mimics the time variables for dynamical systems growing with saturation.

Thus, the desired cumulative distribution function was searched in the form

5 $$\begin{aligned} c.d.f.(A)=\sum \limits _{j=1}^2G_j(A) \end{aligned}$$

with the components consisting of Gompertz functions, which are formally equivalent to Gumbel’s c.d.f’s

6 $$\begin{aligned} G_j(A)=K_je^{-e^{-r_j\left( A-{A_{m}}_j\right) }}, \end{aligned}$$

where $K_j$, $r_j$, and ${A_{m}}_j$ are the maximal value, the growth rate, and the amplitude corresponding to the inflection point, respectively.

The respective probability density components are

7 $$\begin{aligned} P_j(A)=K_jr_je^{-e^{-r_j\left( A-{A_{m}}_j\right) }}e^{-r_j\left( A-{A_{m}}_j\right) }\equiv K_j\frac{1}{r_j^{-1}}e^{-\left[ \left( \frac{A-{A_{m}}_j}{r_j^{-1}}\right) +e^{-\left( \frac{A-{A_{m}}_j}{r_j^{-1}}\right) }\right] }. \end{aligned}$$

Note that Eq. (7) coincides with the Gumbel distribution widely applied in the statistical theory of extreme events^32^. Factors $K_j$ define the normalisation to the value of partial total probability in such a way that only their sum is equal to unity. Respectively, $r_j^{-1}$ and ${A_{m}}_j$ are the scale and the mode of Gumbel-distributed random numbers *A*, their mean value is equal to $E(A)={A_{m}}_j +r_j^{-1}\gamma$, where $\gamma \approx 0.5772$ is the Euler–Mascheroni constant, the standard deviation $\sigma =\pi r_j^{-1}/\sqrt{6}$ and the median is equal to $E(A)={A_{m}}_j +r_j^{-1}\ln \left( \ln (2)\right)$.

An accuracy, with which each component satisfies the Gompertz function, can be easily traced as above again considering the variable alteration stated by the generalized Fisher–Pry transform but applied to each component separately, i.e. which linearizes Eq. (6):

8 $$\begin{aligned} FP(A)\equiv -\ln \left[ \ln \left( \frac{K_j}{A}\right) \right] =r_jA+{A_{m}}_j. \end{aligned}$$

Finally, it should be pointed out that the points indicating “experimental points belonging to different components” are not necessary mutually exclusive data points from the initial dataset. Two of such points may share the same value of the original *A* being taken with weights corresponding to the inputs from particular coexisting random processes, see details in^31^.

**Figure 3:**
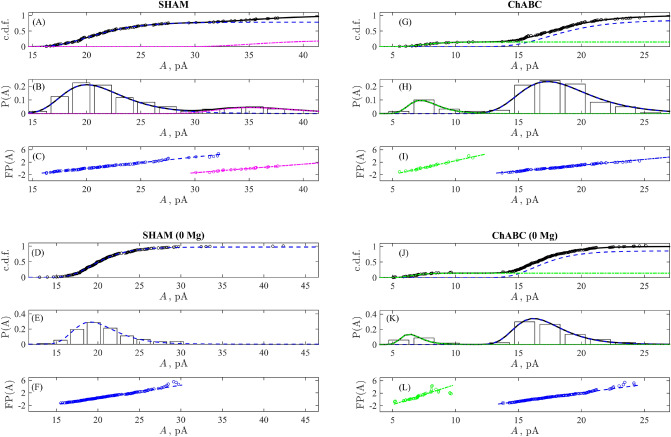
Statistical analysis of mEPSC amplitudes for SHAM (**A**–**C**) and ChABC (**G**–**I**) experimental conditions as well as for NMDA receptor-dependent mEPSCs amplitudes for SHAM 0 Mg (**D**–**F**) and ChABC 0 Mg (**J**–**L**). Empiric c.d.f.’s formed by experimental data are shown as black circles in (**A**,**D**,**G**,**J**) along with their fitting by Eq. 5 (black solid curve) and the individual components (blue dashed and green dash-dotted curves). Subpanels (**B**,**E**,**H**,**K**) demonstrate the respective empiric probability distributions (histograms with bins of the widths $\Delta A=2$ pA centred in semi-integer points), the total composite p.d.f. as a sum of Gumbel’s components given by Eq. 6 (black solid line) and partial weighted Gumbel distributions (blue dashed and green dash-dotted curves). The generalized Fisher–Pry plots (8) for the two revealed components, where solid lines indicated linear fits best approximating the subdivided components of experimental data (circles) in the least-mean square sense are shown in (**C**,**F**,**I**,**L**).

The obtained mEPSCs datasets for four experimental conditions (SHAM, ChABC, SHAM 0 Mg, ChABC 0 Mg) were analyzed according to the procedure described above. The results of the data processing are shown in Fig. 3. The key illustrations are given in subpanels (A,D,G,J) showing an accurate reproduction of the empiric c.d.f. formed by the experimental data with the proposed model Eqs. (5)–(6) containing either two or one Gumbel’s (Gompertz’s) components (their numerical parameters as well as statistical criteria for the goodness-of-fit are provided in Supplementary material). The respective p.d.f. of the components (7) are plotted in subpanels (B,E,H,K) and superimposed for the sake of qualitative comparison with the histograms of the experimental data distributions. To confirm this decomposition quantitatively, Fig. 3C,F,I, and L represent the generalized Fisher–Pry plots (8), from which one can see coinciding linearity of the experimental data and their fitting by the proposed approximation.

Under control conditions in sham solution the mEPSCs amplitude distribution clearly showed two modes (Fig. 3A–C). The first major mode characterised by the mean mEPSC amplitude of 21.6 ± 3.5 pA (mean$\pm$ $\sigma$) giving us estimation of quantal size and variability. The mean amplitude of the second mode for large-amplitude mEPSCs is equal to 37.2 ± 4.6 pA. Several studies support the interpretation that the multiple modes in some of the mEPSC amplitude histograms reflect the spontaneous multivesicular release^10,18–20,33,34^. The differently sized mEPSCs were not due to the chance summation of randomly occurring monoquantal events, because the mEPSC frequency was low (approximately 45 events per minute in 7 cells). In our case large mEPSCs from the second mode of the amplitude distribution seemed to be generated by two vesicles released with small temporal jitter. Thus under this assumption under control conditions the quantal size estimated from mEPSCs amplitude distribution is approximately equal to 21 pA. Changing the extracellular solution by removing Mg$^{2+}$ and addition of AMPA receptor blocker caused the disappearance of the large-amplitude mEPSCs subpopulation (Fig. 3D–F). While the mean NMDA-dependent mEPSCs amplitude remained almost the same as for the main mode of distribution in control and was equal to 20.5 ± 3.1 pA. This finding suggests that the high-amplitude mEPSCs subpopulation undo control condition can be related to AMPA receptor mediated component of the mEPSCs.

Note that in the SHAM 0 Mg dataset we can reliably identify only one component but with the $K_1<1$ as shown in Fig. 3D. At the same time, it is worth noting that an existence of the second component can not be excluded completely because it could lead to underestimating the data collected in the bin centred at $A=29.5$ pA, see Fig. 3E, and the appearance of the hump-like deviation from linearity in the generalised Fisher–Pry plot in Fig. 3F in the same range of amplitudes. However, a limited amount of registered data with large amplitudes does not allow specifying details of the second component in this case.

Interesting that upon enzymatic attenuation of the ECM in hippocampal slices, the amplitude distributions of mEPSCs in pyramidal neurons also clearly showed bimodality in both cases in control for normal solution and for NMDA receptor-dependent mEPSCs in solution without Mg$^{2+}$ (Fig. 3G–L). However, one can observe the appearance of the low-amplitude mEPSCs subpopulation with a mean mEPSCs amplitude of 7.9 ± 1.4 pA (and 6.8 ± 1 pA for NMDA-dependent mEPSCs at 0 mM [Mg$^{2+}$]), which is three times smaller than the quantal size obtained upon control condition. The mean of the main mode of mEPSCs amplitude distribution not significantly decreased compared to sham case and is equal to 18.8 ± 3.4 pA in the presence of Mg$^{2+}$ and to 17.3 ± 2.3 pA for NMDA-dependent mEPSCs in solution without Mg$^{2+}$. The differences between the mean mEPSCs amplitudes for sham and ChABC cases is less than the largest of the pair of $\sigma$ of their distributions. The appearance of low-amplitude mEPSCs subpopulations and the decrease seen in the mean mEPSC amplitudes of the main mode for both normal and NMDA-dependent mEPSCs after ChABC treatment can indicate an increase in the number of new unpotentiated synapses onto CA1 pyramidal neurons following ECM attenuation. This result is consistent with the observation that immature synapses have lower quantal amplitude than potentiated ones^35^.

The results obtained using the proposed analysis are consistent with the findings of the original work^28^, from which we used the mEPSC datasets. In that study, morphological observations based on the 3D reconstruction of electron microscopy images of hippocampal slices revealed that ECM attenuation increases the number of glutamatergic synapses onto CA1 pyramidal neurons. However, the traditional approach of electrophysiological data analysis, based on the comparison of cumulative probability distributions of mEPSC amplitudes and estimating of mean mEPSC amplitudes for the entire dataset, was not able to reveal the appearance of a low-amplitude mEPSC subpopulation upon ECM attenuation. Consequently the shown^28^ statistically significant decrease in the mean mEPSC amplitude upon ECM attenuation does not coincide with the results of our analysis.

## Discussion

In this paper, we present the following key finding: a novel sequential procedure for precise analysis of mEPSC amplitude distributions, which argues in favour of the representation of mPSC amplitudes’ statistics as following the Gumbel distribution, which is directly related to the extreme events theory, which considers the registered spikes as the such events. We have shown that the proposed approach can reliably detect multimodality in the distributions of mEPSC amplitude, allowing for accurate determination of the size and variability of the synaptic response, which is more statistically rich than the simplest quantal picture.

The proposed procedure for mEPSC amplitude distributions analysis does not operate with the fitting of empiric frequencies of the events’ occurrences to some a priory chosen model probability density function since this widely used approach is not robust and leaves a variety of interpretations for different skewed bell-shaped functions. On the contrary, we deal with the cumulative distribution functions, i.e. with the representation known as belonging to the class of robust methods^29,36^ and also provides a possibility to judge the adequacy of the method using the linearising variable alteration without referring to details of the optimization procedure’s implementation.

Figure 2 illustrates this feature for the whole class of distributions, which can be considered as the possible generalization of binomial model, i.e. with the probability of spike-induced current defined by a product of a possible current’s value and its complement to unity (a possibility to not discharge). It is shown that neither of finite integer power laws lead to adequate linearisation of the direct experimental data.

**Figure 4:**
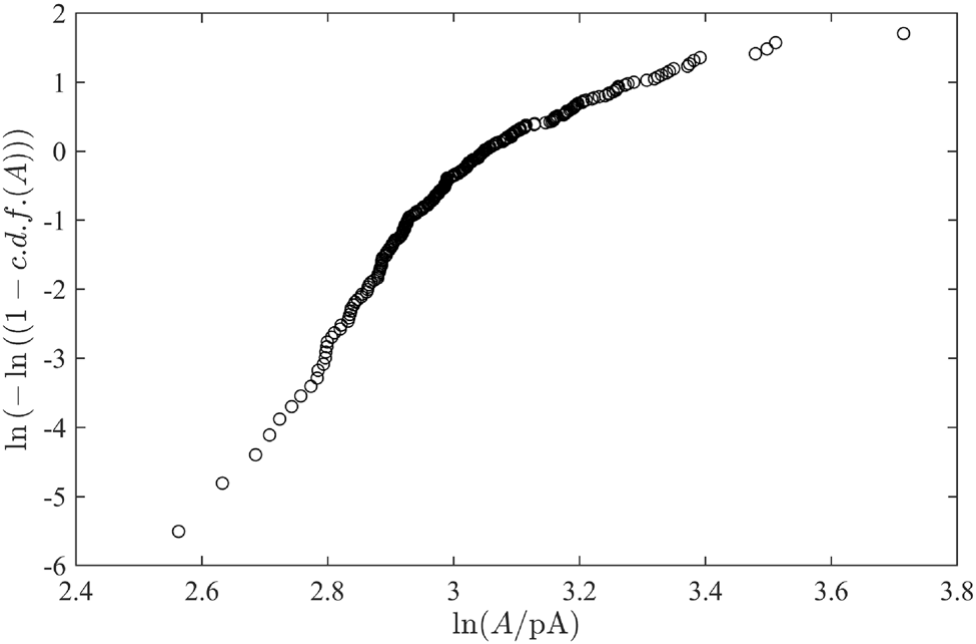
Testing of applicability of the Weibull distribution to the statistics of experimental data recorded in Mg$^{2+}$ free sham solution (SHAM 0 Mg).

Figure 4 illustrates this approach by the case study of testing of the Weibull distribution (as a generalization of the Gamma distribution), which has been discussed earlier^23,27^ as a possible candidates for such statistics. The Weibill distribution has the cumulative distribution function

$$\begin{aligned} {c.d.f.}_{W}(A)=1-\exp \left( -(A/\lambda )^k\right) , \end{aligned}$$

where $\lambda \Gamma \left( 1+1/k\right) =\langle A\rangle$ determines the mean values $\langle A\rangle$ and *k* is the power-law parameter. This c.d.f. allows the linearising variable alteration, see labels of axes in Fig. 4, which directly operates with the recorded data. But is clearly seen that, in contrast to Fig. 3F, it is definitely non a straight line. Thus, the Gumbel distribution demonstrates its indisputable advantage (note that some arguments in favour of such situation were provided in the work^27^ but without such demonstrable unambiguous confirmation).

Another important comparison should be done with the Skew normal distribution (SND), which may originates for the statistics of data obtained, e.g. by the simple artificial elimination of data with amplitudes below some threshold from the full record of normally distributed random process^37^.

**Figure 5:**
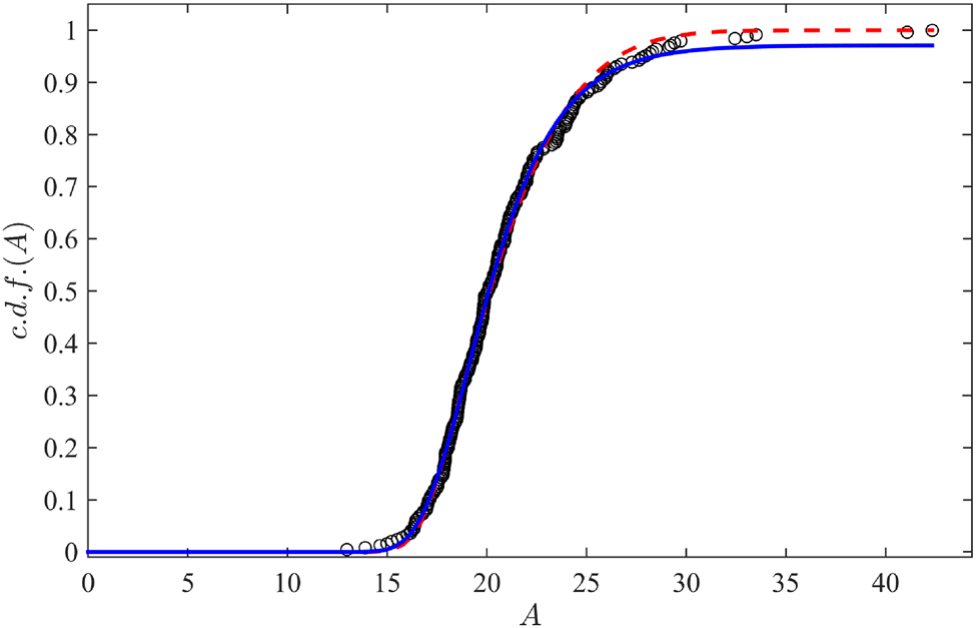
The comparison of the c.d.f. of the Gumbel distribution (solid line) and the Skew normal distribution (dashed line) fitted to the experimental data (circles) recorded in Mg$^{2+}$ free sham solution (SHAM 0 Mg).

In this case, one can see from Fig. 5 that both c.d.f.’s corresponding to the Gumbel distribution [Eq. (6)] and to the SND, which has the form

9 $$\begin{aligned} c.d.f._{SND}(A)=\int \limits _{-\infty }^{\frac{A-A'}{\omega }} \left[ 1+{\mathrm{erf}}\left( \alpha \frac{x}{\sqrt{2}}\right) \right] e^{-\frac{x^2}{2}} \frac{dx}{\sqrt{2}} \end{aligned}$$

have similar reproducibility of the data, although the Gumbel distribution is slightly better: the correlation coefficients are equal to 0.9992 and 0.9950 for the Gumbel distribution and the SND, respectively; the relative average absolute deviations between the experimental and the fit-based data are equal to $AAD_{Gumbel}=0.93\,\%$ and $AAD_{Gumbel}=1.32\,\%$. There are two reasons for this: (1) the SND is more flexible (Eq. 9) has four parameters, $A'$, $\omega$, $\alpha$, for the adjustment if the asymptotic values is strictly equal to one, otherwise their number is equal to five) while the Gumbel-Gompertz expression has only three parameters even taking into account an indefinite asymptotic value; (2) the Gumbel distribution relates to the statistics of extremes in the time series, i.e. it is also a kind of the sample’s truncation although, as it will be discussed below, this variant looks more biophisically-relevant.

The best overlapping two cumulative distributions seen in Fig. 5 is observed in the middle part, which is around the mode of the probability density functions. From Eq. (7),

10 $$\begin{aligned} P(A)= K\frac{1}{r^{-1}}e^{-\left[ \left( \frac{A-{A_{m}}}{^{-1}}\right) +e^{-\left( \frac{A-{A_{m}}}{r^{-1}}\right) }\right] }\approx K\frac{1}{r^{-1}}e^{-\left[ \left( \frac{A-{A_{m}}}{^{-1}}\right) + 1-\left( \frac{A-{A_{m}}}{r^{-1}}\right) +\frac{1}{2}\left( \frac{A-{A_{m}}}{r^{-1}}\right) ^2\right] }= K\frac{1}{r^{-1}}e^{-\frac{1}{2}\left( \frac{A-{A_{m}}}{r^{-1}}\right) ^2}, \end{aligned}$$

i.e. reproduces the Gaussian distribution in the vicinity of $A=A_m$.

The probability density functions corresponding to Eq. (9) has the same property around $A=A'$:

11 $$\begin{aligned} P_{SND}(A)=\left[ 1+{\mathrm{erf}}\left( \alpha \frac{x}{\sqrt{2}}\right) \right] e^{-\frac{x^2}{2}} \frac{1}{\sqrt{2}}\approx \frac{1}{\sqrt{2}}e^{-\frac{1}{2}\left( \frac{A-A'}{\omega }\right) ^2}. \end{aligned}$$

Both expressions, Eqs. (10) and (11), supports the classic idea^3^ that individual quantal peaks are not a pointwise-supported but has a finite wide due to the normal-distributed nose widening. Thus, our results, which operate with the single-quantum range of currents are in line with this hypothesis but refine it taking into account skewness of the distribution due to the specificity of the experiments’ realisation. In addition, we would like to stress again, despite the similarity of Eqs. (10) and (11), the former in the full range of *A* provides a more clear picture.

Let us consider this in more detail. From the computational point of view, the approach proposed in this work, based on the Gompertz equation, and leading the the Gumbel distribution, operates with the model, which allows the linearising variable alteration, see Figs. 2 and 3F, which is independent of any optimisation procedure, it requires the raw experimental data only to ensure the adequacy of the model. For the SND, there is no such transformation in principle, i.e. quality of regression carried out by the more multiparametric curve is determined by the optimization procedure. For example, looking at Fig. 3F, one can see a “hump-like” deviation from linearity for the current amplitude around 28 pA and, respectively, to interpret it as traces of very rare spikes of the higher mode. No such conclusion can be made from the optimization-based procedure for the SND. This is especially important for the case of multimodal signals, see Fig. 3A–C and G–L. Although now the decomposition involves the optimization (not very complicated due to linearizability), the results for both components again can be checked by the existence of linearity for the transformed separated data. There is no such possibility for the SND; moreover, the procedure starts to be more complicated, computationally expensive and less robust due to necessity to deal with numerical integration of Eq. (9) and searching the global minimum influenced by 10 parameters in the case of two components.

The second line of reasoning addresses more biophysical interpretation of experiments and their results. The SND emerges when an observer mechanically cuts-off all data with magnitudes less than some arbitrary threshold. Thus, the rest of the data may include both high-intense components of the background noise and target spikes. On the contrary, the Gumbel distribution emerges when only extreme events for each short subinterval of observations are taken into account. This is namely the procedure of extractions of spikes (i.e. extreme events) from the background electric noise.

This approach provides also a solid statistical background for choosing an appropriate distribution or a combination of superimposed local distributions. When operating with sigmoidal functions representing c.d.f.’s, it is possible to introduce generalised multiparametric functions, which cover a variety of possible distributions and to choose the one, which best resembles the experimental data, and support this choice statistically. As well, processing sigmoidal c.d.f.’s allows for transferring to this new area of applications the methods developed in the field of population dynamics and mathematical economics for the decomposition of a process with multiple saturated states into its constituent components. This transfer is based on the general mathematical similarity of expressions for the saturated growth curves and the asymptotic tending of the cumulative probability distribution functions when time in the former case is replaced with an ordered set of registered values of measured stochastic quantities in the latter.

To illustrate the developed approach, we chose previously obtained experimental data of mEPSCs amplitudes on the effect of the extracellular matrix on synaptic plasticity^28^. Proposed statistical technique allowed us to identify previously unnoticed additional modality in the mEPSC amplitude distribution, indicating the formation of new immature synapses upon ECM attenuation.

Presence of different components in amplitudes of mEPSCs could reveal different actions of ECM removal in Sham and ChABC conditions. Consistently with previous data^28^, the second observed low-amplitude subpopulation of mEPSCs upon ECM attenuation could be mediated by currents occurring in new immature spines, which are not present in Sham. Alternatively, this second component could also be observed as a result of increased in glutamate diffusion after ECM removing^38,39^ and could trigger activation of a larger number of NMDA receptors in ChABC condition. The latter, could significantly affect synaptic transmission and plasticity. Therefore, current approach can help to identify different physiologically relevant components of mEPSCs.

Note that due to the low frequency of mEPSC generation in our analysis, we used the mEPSC amplitude dataset for all recorded cells together. As a result, the second low-amplitude component of the distribution may correspond to outlier cells with measurement artifacts (such as having a large resistance and, accordingly, smaller current amplitudes). However, the proposed statistical procedure can automatically identify and account for such outliers. To resolve this issue, the mEPSC amplitude should be analyzed in individual recordings. The approach proposed in this study allows researchers to automatically identify features of the data that require additional checks and can significantly increase the informativeness of both existing and newly obtained experimental data.

Note also, that identification of different statistical components of mEPSCs may have functional significations for neuronal circuits at system level. Purely random fluctuations with smaller amplitudes were local events determined only by quantal nature of synaptic release. The extreme events required more level of coherence that can be provided by different mechanisms of network or system level activation including neuron circuit signalling and extrasynaptic processes.

We chose to use mEPSC recording data to illustrate the proposed statistical approach because they are the most difficult to analyze and are valuable in determining the quantal response. The problems associated with mEPSC data processing are as follows: (1) data points are noisy and scarce; (2) mEPSCs are highly variable; (3) mPSC amplitudes are small relative to the recording noise; and finally, (4) in practice, mEPSC amplitude histograms are skewed toward larger amplitudes and differ from a normal distribution, making it a challenge to find a continuous function that fits the mEPSC amplitude distributions well. Despite these difficulties, estimates of quantal size and its variability from the mEPSC amplitude distributions are highly sought after, as they are used as a reference for correcting methods for establishing the quantal parameters that describe synaptic transmission and indicate the locus of synaptic plasticity. In addition to mEPSC data, the developed approach could be applied to detect multimodality and precisely estimate the characteristics of different modes in the amplitudes of evoked PSCs. Usually, an amplitude histogram of evoked PSCs demonstrates narrow, well-defined multiple peaks that can be fitted as the sum of several Gaussian curves, with amplitudes corresponding to integral multiples of quantal size. However, there are several potential sources of error in fitting distributions using this approach, such as the dependency of the fitting algorithm on bin size, variations in data subsets, and finite sampling from large populations. Thus, it was essential to subject the amplitude data to various tests to evaluate the relative quality of statistical models for a given dataset. Within this context, the methods of similarity quantification, which is independent of an procedure of optimisation during fitting and operates with the raw data only, look more convincing. The discussed similarity between the Gompertz equation known from the theory of dynamical systems and the Gumbel distribution related to stochastic processes, is a promising example toward the mentione direction.

Finally, we can highlight that the p.d.f.’s for both components belong to the same kind of distribution, which argues in favour of its interpretation as an example of the extreme value process. The “high” values of micro-currents, which reflect spontaneous synaptic activity, are relatively rare events emerging against the background of sub-threshold activity. It is worth noting that this situation is conceptually close to the subject of nonequilibrium statistical physics of small fluctuating systems, where noise plays a significant and constructive role^40,41^. In particular, one can note recent combinatorial arguments^42^ for an emergence of the extreme value statistics in the presence of excited/non-excited states instead of the canonical distribution.

## Methods

### Slice preparation and electrophysiology

All experiments were performed in 4- to 6-week-old C57BL/6J male mice. Animals were killed by cervical dislocation and then decapitated. The brains were exposed and chilled with ice-cold solution containing (in mM) 87 NaCl, 2.5KCl, 7 MgCl$_{2}$, 1.25 NaH$_{2}$PO$_{4}$, 26.2 NaHCO$_{3}$, 0.5 CaCl$_{2}$, 25 D-glucose, and 50 sucrose. Hippocampi from both hemispheres were isolated, and 350 μm-thick transverse slices were cut with a vibrating microtome (Microm HM 650 V, Thermo Fisher Scientific, or VT1200S, Leica). Slices were incubated in a 3-mL chamber for 2 h at 37 $^{\circ }$C in a solution containing (in mM) 113 NaCl, 2.38 KCl, 1.24 MgSO$_{4}$, 0.95 NaH$_{2}$PO$_{4}$, 24.9 NaHCO$_{3}$, 1 CaCl$_{2}$, 1.6 MgCl$_{2}$, 27.8 D-glucose, and 0.2 $\%$ bovine serum albumin (sham) or in the same solution supplemented with 0.2 U/mL of protease-free chondroitinase ABC (ChABC) from Proteus vulgaris (Amsbio, UK). Attenuation of ECM by ChABC was confirmed with a loss of Wisteria floribunda agglutinin (WFA) labeling. Next, the slices were transferred to a recording chamber and were continuously perfused with a solution containing (in mM) 119 NaCl, 2.5KCl, 1.3 MgSO$_{4}$, 1 NaH$_{2}$PO$_{4}$, 26.2 NaHCO$_{3}$, 2.5 CaCl$_{2}$, and 11 D-glucose. All solutions were saturated with 95$\%$ O$_{2}$ and 5$\%$ CO$_{2}$. The osmolarity was 295 ± 5 mOsm.

**Figure 6:**
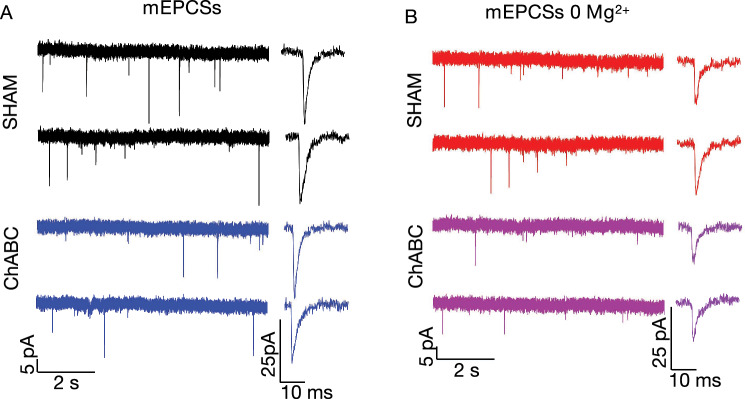
(**A**) Representative traces of mEPCSs in Sham (black) and ChABC (blue). (**B**) Representative traces of NMDA-mediated mEPCSs in Sham (red) and ChABC (magenta).

Whole-cell recordings from CA1 pyramidal neurons were obtained with glass electrodes (3–5 M$\Omega$ resistance). mEPSCs were recorded using an intracellular solution containing (in mM): 130 KCH$_{4}$SO$_{4}$, 8 NaCl, 10 Na phosphocreatine, 10 HEPES, 2 EGTA, 3 Na L-ascorbic acid, 10 HEPES, 0.4 NaGTP, 2 MgATP, and 5 QX314 Br (pH adjusted to 7.2 with KOH and osmolarity adjusted to 290 mOsm). The membrane potential was clamped at − 70 mV. The extracellular solution contained 100 μM picrotoxin, 200 μM (S)-a-methyl-4-carboxyphenyglycine (MCPG), 5 μM CGP52432, and 1 μM tetrodotoxin to block GABAA, mGluR, GABAB receptors, and action potentials, respectively. mEPSCs mediated by NMDA receptors were pharmacologically isolated by applying 25 μM NBQX, an AMPA receptor blocker. NMDA-mediated mEPSCs were recorded using an intracellular solution containing (in mM): 130 KCH$_{3}$SO$_{3}$, 8 NaCl, 10 Na phosphocreatine, 10 HEPES, 2 EGTA, 3 Na L-ascorbic acid, 10 HEPES, 0.4 NaGTP, 2 MgATP, and 5 QX314 Br (pH adjusted to 7.2 with KOH and osmolarity adjusted to 290 mOsm) in Mg$^{2+}$ free extracellular solution in the presence of 100 μM picrotoxin, 200 μM MCPG, 5 μM CGP52432. Figure 6 illustrates representative traces of mEPCSs and NMDA-mediated mEPSCs (recorded at 0 Mg$^{2+}$ and additionally in the presence of AMPARs blocker). From our measurements, we observe significantly lower noise level compared to amplitude of events. The amplitude of mEPCSs and NMDA-mediated mEPSCs were similar for both Sham and ChABC and ranged from average from 15 to 35 pA, however the amplitude was slightly lower in ChABC and ranged from 10 to 25 pA.

Electrophysiological data were analyzed with WinWCP, Mini analysis (6.0.2, Synaptosoft, USA), and Clampfit (9.0 Axon Instruments Inc.; Union City, CA, USA). Detection of mEPSC was done with a first-order derivative algorithm, applied to traces after digital low-pass filtering at 2 kHz.

### Supplementary Information

Supplementary Information.

## Data Availability

Data and code available from the corresponding author on reasonable request.
