# Supplementary material for: Estimation of cumulative amplitude distributions of miniature postsynaptic currents allows characterising their multimodality, quantal size and variability
Source: Sci Rep. 2023 Sep 20;13:15660. doi: 10.1038/s41598-023-42882-9 (PMC10511413; doi:10.1038/s41598-023-42882-9)
Supplement: Supplementary file 1 — Supplementary Information. [file 41598_2023_42882_MOESM1_ESM.pdf]

Supplementary material for
“Estimation of Cumulative Amplitude Distributions of Miniature Postsynaptic Currents Allows Characterising their Multimodality, Quantal Size and Variability”

Table 1. Parameters of the component - Gompertz functions (Eq.6), which fit the cumulative distribution functions of the mEPSCs amplitudes for Sham condition (Fig. 3 (A-C)) and the respective Gumbel distribution of spikes.

Gompertz		*K*	A_m	*r*	mean	σ	median	SSE	RMS	MAD	MAPE	SE	ln[MLE]	AICc	R^2	*p value*
Sham control	Component 1●	0.786	20.0	0.366	21.577	3.5	18.99	0.0173	0.0128	0.0101	0.0511	0.0130	308	-610	0.993	8.04e-112
	Component 2●	0.214	35.1	0.276	37.191	4.6	33.77	0.000596	0.00546	0.00460	0.122	0.00592	75.8	-144	0.977	2.98e-16
	Whole fit							0.0178	0.0122	0.00942	0.0453	0.0125	359	-705		

Table 2. Parameters of the component - Gompertz functions (Eq.6), which fit the cumulative distribution functions of the mEPSCs amplitudes for ChABC control condition (Fig. 3 (G-I)) the respective Gumbel distribution of spikes.

Gompertz		*K*	A_m	*r*	mean	σ	median	SSE	RMS	MAD	MAPE	SE	ln[MLE]	AICc	R^2	*p value*
ChABC control	Component 1●	0.149	7.22	0.888	7.87	1.4	6.81	0.000429	0.00463	0.00359	0.238	0.00503	79.1	-151	0.977	2.63e-15
	Component 2●	0.851	17.3	0.375	18.84	3.4	16.32	0.0341	0.0185	0.0151	0.0724	0.0187	257	-508	0.990	2.83e-99
	Whole fit							0.0345	0.0170	0.0132	0.0679	0.0174	319	-625		

Table 3. Parameters of the component - Gompertz functions (Eq.6), which fit the cumulative distribution functions of the mEPSCs amplitudes for Sham 0 Mg condition (Fig. 3 (D-F)) the respective Gumbel distribution of spikes.

Gompertz		*K*	A_m	*r*	mean	σ	median	SSE	RMS	MAD	MAPE	SE	ln[MLE]	AICc	R^2	*p value*
Sham 0 Mg	Component 1●	0.971	19.1	0.410	20.51	3.1	18.21	0.0291	0.0111	0.00870	0.0351	0.0112	724	-1441	0.912	1.25e-123

Table 4. Parameters of the component - Gompertz functions (Eq.6), which fit the cumulative distribution functions of the mEPSCs amplitudes for ChABC 0 Mg condition (Fig. 3 (G-I)) the respective Gumbel distribution of spikes.

**Gompertz**		***K***	***A_m***	***r***	**mean**	**σ**	**median**	**SSE**	**RMS**	**MAD**	**MAPE**	**SE**	**ln[MLE]**	**AICc**	**R^2**	***p value***
**ChABC 0 Mg**	**Component 1**●	0.857	16.2	0.545	17.26	2.3	15.53	0.0249	0.0109	0.00940	0.0415	0.0110	644	-1282	0.913	6.89e-110
	**Component 2**●	0.143	6.31	1.30	6.754	0.99	6.03	0.00501	0.0113	0.00887	0.831	0.0118	119	-231	0.859	2.60e-14
	**Whole fit**							0.0319	0.0112	0.00948	0.250	0.0113	780	-1548		

K, *r*, and A_m are the maximal value, a kind of the "growth rate", which characterizes the growth the cumulative distribution function as a function of mEPSCs amplitudes**,** and the amplitude corresponding to the inflection point, respectively; "mean" and σ are the mean value and the standard deviation of the corresponding Gumbel distribution. Goodness of fit determined by the following parameter values: the sum of squares (SSE), root mean square (RMS), mean absolute deviation (MAD), the mean absolute percentage error (MAPE), Standard Error of the fit (SE), maximum likelihood estimation (MLE), the corrected Akaike information criterion for model selection (AICc; Cavanaugh 1997), coefficient of determination (R^2) and *p*-value.

References

Cavanaugh JE. 1997. Unifying the derivations for the Akaike and corrected Akaike information criteria. Statistics & Probability Letters, 33(2);201-208. doi:10.1016/S0167-7152(96)00128-9
